# In Situ Regolith Seismic Velocity Measurement at the InSight Landing Site on Mars

**DOI:** 10.1029/2022JE007229

**Published:** 2022-09-30

**Authors:** Nienke Brinkman, Cédric Schmelzbach, David Sollberger, Jan ten Pierick, Pascal Edme, Thomas Haag, Sharon Kedar, Troy Hudson, Fredrik Andersson, Martin van Driel, Simon Stähler, Tobias Nicollier, Johan Robertsson, Domenico Giardini, Tilman Spohn, Christian Krause, Matthias Grott, Jörg Knollenberg, Ken Hurst, Ludovic Rochas, Julien Vallade, Steve Blandin, Philippe Lognonné, W. Tom Pike, W. Bruce Banerdt

**Affiliations:** ^1^ Institute of Geophysics ETH Zürich Zürich Switzerland; ^2^ NASA Jet Propulsion Laboratory California Institute of Technology Pasadena CA USA; ^3^ Eidgenössische Forschungsanstalt WSL Birmensdorf Switzerland; ^4^ Deutsches Zentrum für Luft‐ und Raumfahrt (DLR) Bremen Germany; ^5^ International Space Science Institute Bern Switzerland; ^6^ Centre National des Études Spatiales (CNES) Toulouse France; ^7^ Université Paris Cité Institut de physique du globe de Paris CNRS Paris France; ^8^ Imperial College London UK

## Abstract

Interior exploration using Seismic Investigations, Geodesy and Heat Transport's (InSight) seismometer package Seismic Experiment for Interior Structure (SEIS) was placed on the surface of Mars at about 1.2 m distance from the thermal properties instrument Heat flow and Physical Properties Package (HP^3^) that includes a self‐hammering probe. Recording the hammering noise with SEIS provided a unique opportunity to estimate the seismic wave velocities of the shallow regolith at the landing site. However, the value of studying the seismic signals of the hammering was only realized after critical hardware decisions were already taken. Furthermore, the design and nominal operation of both SEIS and HP^3^ are nonideal for such high‐resolution seismic measurements. Therefore, a series of adaptations had to be implemented to operate the self‐hammering probe as a controlled seismic source and SEIS as a high‐frequency seismic receiver including the design of a high‐precision timing and an innovative high‐frequency sampling workflow. By interpreting the first‐arriving seismic waves as a P‐wave and identifying first‐arriving S‐waves by polarization analysis, we determined effective P‐ and S‐wave velocities of $v_{P}=119_{-21}^{+45}$ m/s and $v_{S}=63_{-7}^{+11}$ m/s, respectively, from around 2,000 hammer stroke recordings. These velocities likely represent bulk estimates for the uppermost several 10s of cm of regolith. An analysis of the P‐wave incidence angles provided an independent *v*_*P*_/*v*_*S*_ ratio estimate of $1.84_{-0.35}^{+0.89}$ that compares well with the traveltime based estimate of $1.86_{-0.25}^{+0.42}$. The low seismic velocities are consistent with those observed for low‐density unconsolidated sands and are in agreement with estimates obtained by other methods.

## Key Points

- Seismic signals from the Heat flow and Physical Properties Package mole provide a unique opportunity to study the shallow regolith
- First‐arrival traveltimes and P‐wave incidence angles constrain elastic parameter estimates
- Low seismic velocities are consistent with unconsolidated low‐density sand

## 1 Introduction

**Figure 1:**
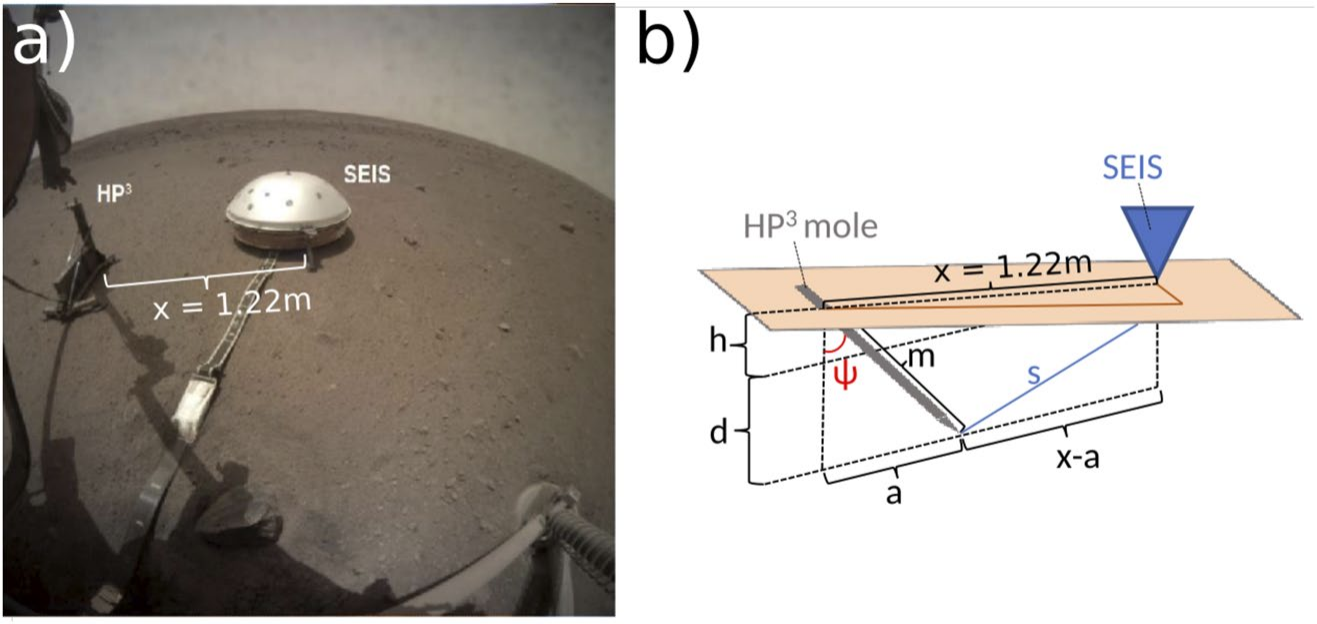
(a) Image showing both the Heat flow and Physical Properties Package (HP^3^) and Seismic Experiment for Interior Structure (SEIS) instruments at the InSight landing site on Mars. (b) Schematic illustration of the HP^3^ and SEIS geometry. The blue triangle marks SEIS, while the 40‐cm long HP^3^ mole is displayed in gray. The orange surface represents the slightly tilted Martian surface. Variables are explained in Section 3.2.

The NASA InSight (Interior exploration using Seismic Investigations, Geodesy and Heat Transport) lander touched down at Elysium Planitia on Mars in November 2018. The main goal of the mission is to investigate the internal structure of Mars using seismic, geothermal, and radio science experiments (Banerdt et al., 2020). Two scientific instruments were deployed on the surface of Mars (Figure 1a): (a) The Seismic Experiment for Interior Structure (SEIS) package (Lognonné et al., 2019) that consists of two three‐component seismometers to monitor the Martian seismicity (e.g., Clinton et al., 2021; Giardini et al., 2020) and to image the interior of the planet (e.g., Khan et al., 2021; Knapmeyer‐Endrun et al., 2021; Lognonné et al., 2020; Stähler et al., 2021) and (b) the Heat flow and Physical Properties Package (HP^3^; e.g., Grott et al., 2021; Spohn et al., 2018), serving the purpose of determining the heat budget of the planet via heat flow measurements at various depths. A self‐hammering probe (hereinafter referred to as the mole) is included in the HP^3^ package and was designed to penetrate into the Martian subsurface to acquire heat flow measurements down to a depth of three to five m.

The hammering of the mole‐generated distinct seismic signals that were recorded by SEIS: These signals provide a unique opportunity to study the elastic parameters of the very shallow subsurface at the InSight landing site. Estimates of the seismic velocities provide insights into the composition and state of the shallowest regolith layer (i.e., the unconsolidated surface layer primarily formed by meteorite impacts over geological time) that are relevant for studying the local geology (e.g., aeolian processes and deposition history), understanding the coupling of SEIS to the ground, constraining other seismic investigations, and providing critical geotechnical parameters for future missions.

Seismic experiments to image the shallow subsurface have been performed on the Moon during the Apollo missions 14, 16, and 17. The data analysis is still ongoing and keeps revealing new information about the lunar subsurface (e.g., Cooper et al., 1974; Heffels et al., 2017, 2021; Larose et al., 2005; Sollberger et al., 2016). More recently, the seismic analysis of the MUPUS hammering signals during the Rosetta mission enabled inferring the elastic parameters of the snow and regolith cover on comet 67P/Churyumov‐Gerasimenko (Knapmeyer et al., 2016, 2018; Spohn et al., 2007, 2009, 2015). The MUPUS hammer was located about 1 m away from accelerometers mounted on the lander that recorded the seismic hammering signals. Interestingly enough, this seismic experiment is similar in terms of source type and geometry to the setup of SEIS recording the seismic signals generated during HP^3^ mole hammering.

Seismic investigations of the shallow subsurface at the InSight landing site to date include an initial traveltime analysis of the first HP^3^ hammering sessions (Lognonné et al., 2020), compliance studies (Kenda et al., 2020; Lognonné et al., 2020; Murdoch et al., 2021; Onodera, 2022), and ambient vibrations Rayleigh wave ellipticity inversions (Carrasco et al., 2021; Hobiger et al., 2021). These initial seismic results revealed a low velocity layer (*v*_*P*_ < 300 m/s and *v*_*S*_ < 150 m/s) at the top of the regolith layer that cannot be thicker than 1–1.5 m (Hobiger et al., 2021; Lognonné et al., 2020). These measured low seismic velocities are consistent with the observed impact‐fragmented regolith dominated by sand‐sized unconsolidated particles (Golombek, Warner, et al., 2020) and compare well to laboratory estimates from Mars regolith simulants by Delage et al. (2017). Below 1–2 m depth, the fine‐grained sand appears to be mixed with blocky ejecta, which likely leads to an increase in bulk seismic velocities (*v*_*P*_ > 700 m/s and *v*_*S*_ > 400 m/s) as proposed based on the Rayleigh wave analysis and compliance inversions (Hobiger et al., 2021; Kenda et al., 2020; Lognonné et al., 2020; Onodera, 2022). From the interpretation of orbital images of craters close to the InSight landing site, it was suggested that the regolith layer is around 3–5 m thick on top of a meter to ten‐m‐thick layer of coarse blocky ejecta situated on top jointed basaltic lava flows (Golombek et al., 2017; Warner et al., 2017). Below around 20 m depth, Hobiger et al. (2021) found, based on a Rayleigh wave ellipticity inversion, a sequence of shallow high‐velocity Amazonian age basalt flows, followed by a low‐velocity zone interpreted as a sedimentary layer at 30–75 m depth laying above older Amazonian or Hesperian age basalt flows. A deep sedimentary layer has been proposed at around 175 m depth (Hobiger et al., 2021; Pan et al., 2020).

The recording of the HP^3^ hammering signals with SEIS marks the first controlled‐source seismic experiment on Mars, and the first opportunity to directly measure the seismic velocities of the shallow Martian regolith in situ. The traveltimes of the seismic waves can be used to infer the seismic velocities of the regolith provided that the hammering (source) times can be linked accurately enough with the recording times and that the seismic signals can be recorded with sufficiently high temporal resolution. However, SEIS was primarily designed to record low‐frequency (<1 Hz) marsquakes, and a direct link between the HP^3^ and SEIS clock for time correlation was not foreseen. In this paper, we outline the steps that were necessary to record high‐resolution seismic data in sufficient temporal resolution and accuracy to estimate the regolith P‐ and S‐wave velocities of around 119 and 63 m/s, respectively. Complementary *v*_*P*_/*v*_*S*_ estimates derived from the incidence angle of the first‐arriving P‐waves largely confirm the traveltime‐based results.

## 2 Preparation of the Seismic Recording of the HP^3^ Hammering

Based on prelanding laboratory measurements using Martian regolith simulants, low seismic velocities in the range of around 100 m/s for P‐waves were suggested by Morgan et al. (2018) for the shallowest regolith at the InSight landing site. These low velocity values would result in traveltimes of several milliseconds to around 10 ms for P‐waves at a distance of 1.2 m between the mole acting as seismic source and SEIS. Considering SEIS' shortest nominal sampling interval of 10 ms, it became clear that high‐precision traveltime measurements and a subsequent velocity determination were not possible with these nominal SEIS acquisition settings.

Inferring the regolith seismic velocities thus required addressing questions such as the following:

- Can SEIS, with its sensor and electronics designed to record low‐amplitude and low‐frequency marsquakes, be used to record high‐amplitude and high‐frequency hammering signals?
- Can the hammering time (source time) be determined accurately enough, considering that the hammering time accuracy was of minor importance for the nominal HP^3^ operation?
- How can the hammering times be correlated with the SEIS recordings, considering that a link between HP^3^ and SEIS clocks was not foreseen?
- How does the mole, designed to convert its hammering energy into downward motion and plastic deformation, work as a seismic source? What do the emitted seismic signals look like?

Addressing these questions for the implementation of the experiment involved a series of numerical, laboratory, and analog field tests on Earth and preparatory measurements on Mars (for a comprehensive summary of all prelanding preparatory activities see Kedar et al., 2017).

### 2.1 InSight's HP^3^ Mole and SEIS Instruments

The HP^3^mole is a 40 cm long and 0.85 kg heavy self‐hammering device (Spohn et al., 2018). An electric‐mechanic system consisting of masses and springs was designed to drive the mole downward with repeated hammer blows. Numerical modeling of hammer strokes to study the interaction between the mole mechanism and the surrounding regolith revealed that the mole releases most seismic energy at its tip during forward motion (Lichtenheldt et al., 2014). A total stroke energy of around 0.7 J was measured in the laboratory for a hammer strike with regular mole operation (Wippermann et al., 2020). Estimates of the seismic energy radiated by the mole during one of the first hammering sessions (sol 158) are around 1.3 mJ (Spohn et al., 2021). Hence, only a small portion of the stroke energy was partitioned into seismic energy that reached SEIS. Additionally, a significant portion of the energy was potentially lost due to poor coupling of the mole to the ground and/or significant absorption of energy during propagation through loose material (Prasad et al., 2004).

Seismic data on Mars are acquired using the SEIS package, an assembly of two instruments that includes (a) the very broad band (VBB) and (b) the short period (SP) seismometer designed to record signals in bandwidths from 0.01 to 5 Hz and 0.1–50 Hz, respectively (see Figure 1a; Lognonné et al., 2019). While the velocity output of the VBB rolls off at a corner frequency of around 10 Hz, the velocity output of the SP is flat between 0.0286 Hz and 2 kHz, making the SP the sensor of choice for high‐frequency recording. Additionally, the clipping level of the SP for the gain settings used during all hammering sessions was 0.9 mm/s and was not exceeded during mole hammering, whereas the VBB saturation level of 0.3 mm/s in the 0.05–10 Hz was exceeded a few times during mole hammering.

The acquisition electronics of SEIS, termed the E‐Box, is used to control the two seismometers and record seismic data (Zweifel et al., 2021). The E‐Box was designed to record digital seismic data with a maximum nominal sampling frequency of 100 Hz (i.e., with a sampling interval of 10 ms). Programmable digital finite impulse response (FIR) filters are used as low‐pass (anti‐alias) filters before down‐sampling. Even though it was not a mission design requirement, new FIR filters can be uploaded to the InSight lander from Earth even during mission operation, which turned out to be critical for the successful recording of the hammering.

### 2.2 Time Keeping and Clock Correlation

Measuring seismic wave traveltimes requires the time of the mole impacts (source time) to be known accurately relative to a common time base. Two two‐axis accelerometers provide access to mole inclination information after each stroke to track the mole's movement. Premission tests have been performed at JPL to test the voltage output of the accelerometers before and after hammer strokes in order to determine a threshold value. Once this threshold value is reached, the inclination of the mole can be determined by reading out the voltages of the accelerometers. Three different environmental conditions have been tested to obtain a threshold value that would suit for measurements recorded on Martian ground. Yet, due to disturbed measurement recordings from the first few hammer sessions, which indicate a different Martian environment than previously expected, the predetermined threshold value needed to be manually adjusted (by telecommand). Readouts of the attitude measurements are taken exactly 1 s after threshold value in the acceleration signal is exceeded. These accelerometer measurements, hence, provide an indirect time stamp of each stroke in HP^3^ clock time. The time stamps were stored by the HP^3^ electronics with a sampling frequency of 600 Hz, resulting in a maximum quantization error of the hammer time of 1.67 ms.

Because SEIS and HP^3^ are not connected via a direct communication line, a correlation of the time stamps of the individual measurements had to be performed between their respective internal clocks via the lander clock. The lander and HP^3^ clock have a very high resolution of 1/2^16^ s, whereas the SEIS clock operates with a lower time resolution of 1/2^10^ s (Zweifel et al., 2021). Consequently, the quantization of the SEIS time can introduce an additional time uncertainty of up to around 1 ms when comparing clock readings.

**Figure A1:**
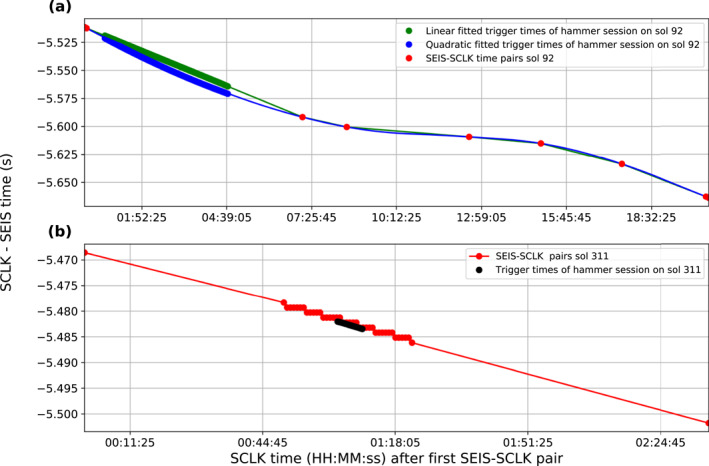
(a) Time correlation pairs (red) for the hammer session conducted on sol 92. The trigger times (block dots) for the hammer sessions are shown in green and blue to illustrate a linear and quadratic behavior between the Seismic Experiment for Interior Structure clock and spacecraft clock kernel, respectively. (b) Time correlation pairs (red) and trigger times (black dots) for the hammer session conducted on sol 311. The time axes for both panels (a and b) are relative to the first available time correlation pair.

A further source of SEIS clock time errors is the nonlinear drift of the SEIS clock that is controlled by the pronounced temperature variations on Mars (Zweifel et al., 2021). To correct for the drift of the SEIS clock relative to the lander clock, correlation pairs (simultaneous time read‐outs from both the SEIS and lander clock) are taken at intervals on the order of hours. Reconstruction of the clock time between correlation pairs taken with hour‐long intervals results in potential clock time differences between the true and reconstructed SEIS time on the order 10's of milliseconds due to the nonlinear nature of the drift (see Figure A1). While such clock time errors are acceptable for low‐frequency marsquake recordings, this clock error is on the order of, or even exceeds the expected HP^3^‐SEIS traveltimes. To address these problems, we therefore implemented a new clock correlation scheme between the lander and SEIS based on 50 s intervals to ensure a negligible SEIS clock correlation error of around 100 μs (i.e., around 1% of the expected traveltime of around 10 ms). A detailed description of the clock‐correlation procedures is given in Appendix A.

### 2.3 High‐Resolution Recording of the HP^3^ Mole Seismic Signals

Experiments with analog mole models were carried out on Earth (both in the laboratory and in the field) to estimate the seismic signature of the mole. These measurements showed that the hammering signals are broadband (Kedar et al., 2017) with dominant frequencies >100 Hz exceeding the highest nominal Nyquist frequency of SEIS of 50 Hz. To address this issue, we designed a new SEIS acquisition flow to exploit the full bandwidth of the seismic signals generated by the mole to resolve the traveltimes at a resolution finer than the nominal sampling interval of 10 ms (Sollberger et al., 2021).

We omitted the nominal low‐pass (anti‐aliasing) FIR filter in the acquisition chain when down‐sampling from 500 to 100 Hz sampling frequency, which results in the seismic data being aliased after down‐sampling (see Appendix B for a detailed description of the implementation, Sollberger et al., 2021). These aliased data contain energy in the frequency range 0–250 Hz but folded around the nominal Nyquist frequency of 50 Hz. To recover the broadband information, Sollberger et al. (2021) developed a de‐aliasing algorithm that is based on the observation that the seismic data of each hammering session contain a high (>20) number of repeated hammer signals with only minor waveform variations between hammer strokes. These waveforms are each subsampled at different points in time because the SEIS sampling process runs independently of the HP^3^ mole hammering timing. Enforcing a sparsity constraint on a Radon transform representation of the signal then enabled us to reconstruct the 0–250 Hz broadband recordings.

### 2.4 Preparatory Measurements on Mars

A series of preparatory test measurements were performed on Mars after landing but before the first hammering session took place. The motivation for these experiments was to test the newly designed SEIS acquisition flow and to address concerns that the high‐frequency band above 50 Hz could be contaminated by strong winds (Teanby et al., 2017), mechanical resonances and SEIS rotation (Fayon et al., 2018), and/or excessive electronic and instrument noise (Zweifel et al., 2021). Measurements with acquisition settings to record information between 50 and 80 Hz showed that ambient noise (e.g., wind‐induced and lander‐induced noise) dominates up to around 60–70 Hz depending on wind conditions (Hurst et al., 2021). Spurious resonances of the SEIS leveling system were observed at 51 Hz (Lognonné et al., 2020) but were later found to be too weak to contaminate the hammering measurements. Above around 60 Hz, the recordings at quiet times are best explained by random noise with an amplitude increase proportional to frequency (in Volt or velocity) as was expected for the acquisition noise (i.e., instrument and electronic noise; Lognonné et al., 2019; Zweifel et al., 2021). Nevertheless, the acquisition noise was later found to be around 30 dB lower in amplitude than the hammering signals, even at the high‐end of the frequency band of interest (i.e., around 120 Hz).

## 3 Acquisition of SEIS Data During HP^3^ Hammering

### 3.1 Time Line of Hammering Sessions

Following the successful deployment of the HP^3^ support system assembly on Mars, the mole hammering operations started at the end of February 2019 on sol 92. Immediately after the first hammer session, it became clear that the mole did not penetrate as planned. Almost a full Martian year (22 months) was devoted to resolving this anomaly. Various attempts were made to assist the mole in penetrating deeper. After realizing that imminent success was not to be expected, the InSight team stopped all efforts to further penetrate the mole in early January 2021 (sol 745), leaving the mole tip buried at a depth of 40 cm (for a comprehensive discussion see Spohn et al. (2021)).

In total, 30 hammer sessions were performed on Mars. Twenty‐seven sessions were recorded by SP using the high‐resolution FIR filter setting, out of which 25 were acquired with a sufficient number of strokes (>20) to be reliably de‐aliased following Sollberger et al. (2021) (Table 1). The hammer sessions conducted on sols 118 and 158 were recorded with improper HP^3^ mole timing settings that caused a large scatter of the source time, leaving 23 hammer sessions with a total of 2,461 hammer stroke recordings for the analysis reported in this paper.

**Table 1:** Overview of All 30 HP^3^ Hammer Sessions Conducted on Mars

Hammer session	Sol	Number of strokes	Cumulative number of strokes	High‐resolution SP FIR filter setting	Used for HP^3^‐SEIS
1	92	3,881	3,881	No	No
2	94	4,720	8,601	No	No
3	118	197	8,798	Yes	No
4	158	198	8,996	Yes	No
5	308	20	9,016	Yes	No
6	311	101	9,117	Yes	Yes
7	315	101	9,218	Yes	Yes
8	318	152	9,370	Yes	Yes
9	322	50	9,420	Yes	Yes
10	325 a	152	9,572	Yes	Yes
11	325 b	152	9,724	Yes	Yes
12	346	40	9,764	Yes	Yes
13	349	50	9,814	Yes	Yes
14	366	19	9,833	Yes	No
15	373	127	9,960	Yes	Yes
16	380	126	10,086	Yes	Yes
17	407	151	10,237	Yes	Yes
18	458	24	10,261	Yes	Yes
19	472	24	10,285	Yes	Yes
20	489	50	10,335	Yes	Yes
21	509	100	10,435	Yes	Yes
22	523	100	10,535	Yes	Yes
23	536	151	10,686	Yes	Yes
24	543	100	10,786	Yes	Yes
25	550	126	10,912	Yes	Yes
26	557	151	11,063	Yes	Yes
27	618	101	11,164	Yes	Yes
28	632	101	11,265	Yes	Yes
29	645	252	11,517	Yes	Yes
30	745	506	12,023	No	No

*Note.* Note that not all sessions conducted with the high‐resolution short period (SP) acquisition settings could be used for the traveltime analysis but only those denoted as “Used for HP^3^‐SEIS”: “Yes.” Cumulative number of strokes refers to the end of each session. See also Spohn et al. (2021).

### 3.2 Acquisition Geometry

**Figure 2:**
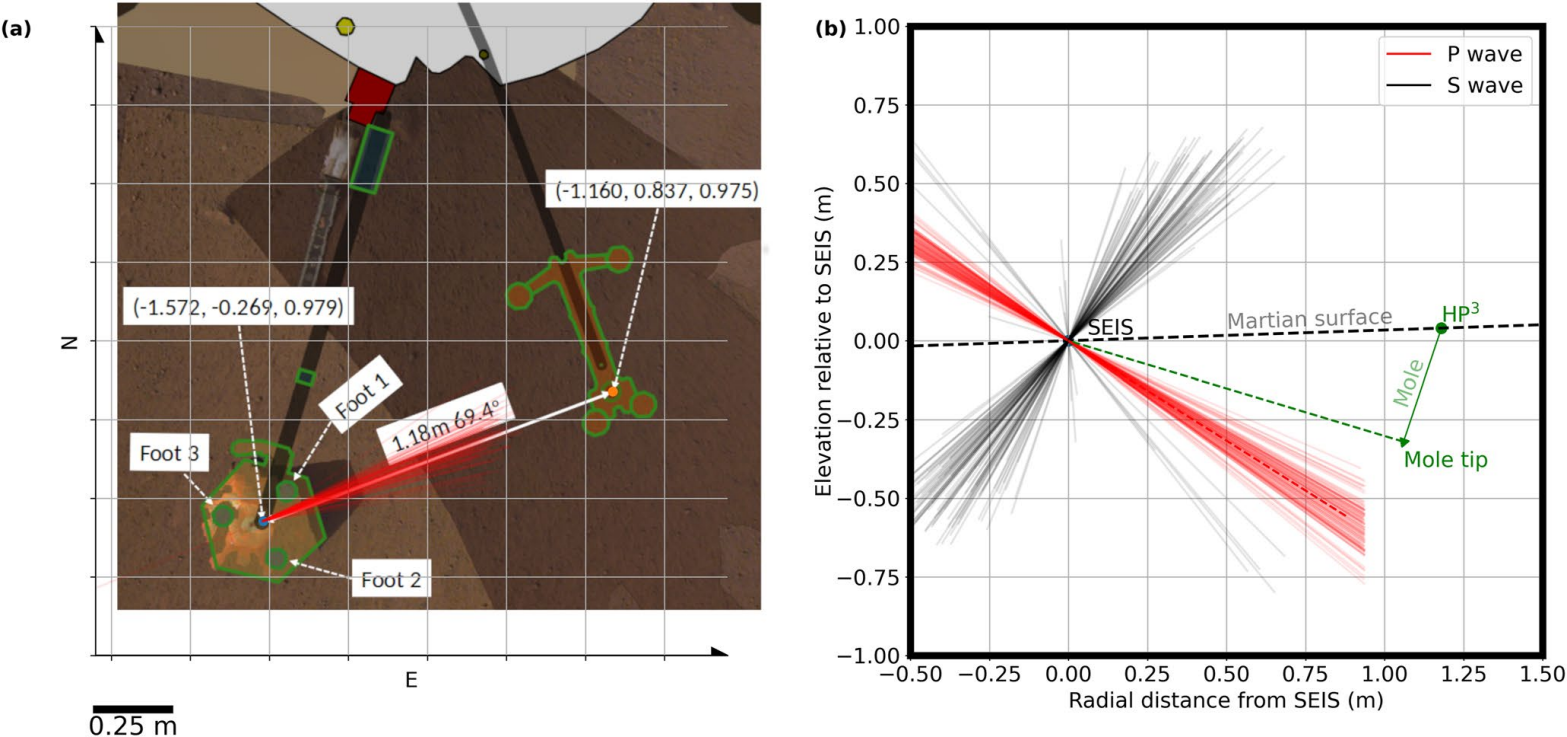
(a) Graphical representation of Heat flow and Physical Properties Package (HP^3^; right), Seismic Experiment for Interior Structure (SEIS) (left), and lander (top) seen from above (image is oriented toward North; see also Figure 1 and Table 2; Golombek, Williams, et al., 2020). Red lines emanating from SEIS with an average azimuth of 69.4° measured clockwise from North (vertical axis) mark the projection of the first‐arrival polarizations eigenvector ${\hat{v}}_{1}$ onto the horizontal plane for the sol‐311 hammering session. (b) View of the vertical plane through HP^3^ and SEIS. Red and black lines show the ${\hat{v}}_{1}$‐ and ${\hat{v}}_{2}$ components first‐arrival polarization eigenvectors, respectively, projected onto this plane. The observed average P‐wave incidence direction (dashed red line) is steeper than the direct mole tip—seismometer line (dashed green line), potentially due to the effect of the free surface on the polarization of the P‐wave (further discussed in Appendix C). We interpret the red and black lines in panels (a and b) as first‐arrival P‐ and S‐wave polarization direction, respectively.

After deployment, the center of the SEIS assembly and the HP^3^ mole egress point were separated by a horizontal and vertical distance of *x* = 1.22 m and *h* = 18 mm, respectively, as determined from high resolution images taken with the two cameras on the InSight lander with an accuracy of about 1 mm (see Figures 1b and 2, Table 2).

**Table 2:** Coordinates of the Seismic Experiment for Interior Structure (SEIS) Assembly Center and the HP^3^ Mole Egress Point in a Local, Right‐Handed (*Z* Positive Downward Along the Gravity Vector) Coordinate Frame With the Origin at the Base of the Shoulder Joint of the Robotic Arm on the Lander Deck

Instrument	*N*‐coordinate (m)	*E*‐coordinate (m)	*Z*‐coordinate (m)
SEIS	−1.5733	−0.2955	0.9957
HP^3^ mole egress point	−1.1361	0.8538	0.9776

*Note.* See Figures 1 and 2a for orientation.

During the hammering sessions, the motion of the mole was tracked using the tilt meters incorporated in the mole and images from the two cameras. The depth of the mole was determined with an accuracy of ±0.5 cm for the hammer sessions on sols 308 to 458 (sessions 5–18), when the mole could be seen by both the cameras on the robotic arm and the lander and later with an accuracy of ±1.0 cm, when the mole could only be imaged from the lander (Spohn et al., 2021). Since the back cap of the mole was flush with the surface after sol 536, the depth of the mole could no longer be determined from camera images and no other means were available to measure the depth of the mole. Hence, no depth readings are available for sessions recorded after sol 536. But, the analysis of images taken during subsequent hammerings indicates that the mole did not significantly move after sol 536 (Spohn et al., 2021).

Given the HP^3^ and SEIS geometry displayed in Figures 1 and 2, the distance *s* between the mole tip and SEIS is defined as (see also sketch in Figure 1b) follows:

(1) $$s=\sqrt{d^{2}+{\left(x-a\right)}^{2}},$$

where *d* is the depth of the mole tip below the level of SEIS, *x* = 1.22 m is the horizontal distance between SEIS and HP^3^, and *a* = *m* sin *ψ* with *m* marking the part of the 40‐cm long mole that is inside the ground and *ψ* denoting the mole tilt angle (measured from vertical). The mole accumulated a tilt *ψ* of about 20° after the first two hammering sessions on sols 92 and 94 with the mole pointing into the direction of SEIS as illustrated in Figure 1b. During subsequent hammering sessions, *ψ* increased further to about 30°.

For the sessions of interest for this study, the mole penetrated from being about halfway buried in the subsurface to a stage where the back cap was completely flush with the regolith. This motion resulted in a reduction of the distance between the mole tip and SEIS *s* from 1.17 to 1.08 m. However, most of the mole motion took place during seven sessions (i.e., sessions on sols 325, 349, 373, 380, 407, 458, and 472) when the mole moved on average >0.16 mm/stroke (Spohn et al., 2021).

### 3.3 High‐Resolution Seismic Waveform Data

**Figure 3:**
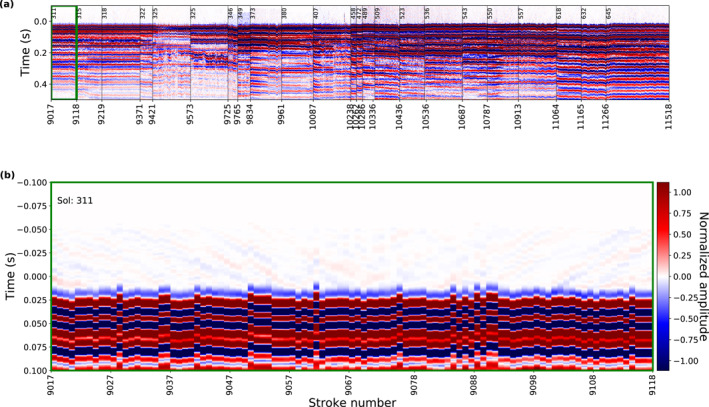
High‐resolution vertical component short period data for all analyzed hammer sessions. (a) Recordings sorted by sequential hammer stroke number (see Table 1) with time relative to the trigger time. The hammer sessions are separated by vertical lines and annotated by the sol when they were recorded. (b) Zoom in on the hammer session conducted on sol 311. The same color‐scale as Figure (b) is used.

Vertical‐component seismic waveform data of all HP^3^ hammering sessions considered in this study are displayed in Figure 3 (see also overview in Table 1). These data were recorded with the high‐resolution acquisition settings on the SP sensor and reconstructed following Sollberger et al. (2021). The time axis in Figure 3a shows time relative to the mole trigger time (corresponding to *t* = 0) after converting the HP^3^ time stamps to SEIS clock time (see Appendix A).

Overall, the waveform data look similar in characteristics within a session but changes between different sessions are noticeable. We suspect that variations in the coupling of the mole to the ground as well as changes in the orientation of the mole relative to SEIS are responsible for these waveform variations.

The first arrivals can be identified several milliseconds after the mole trigger time (see zoom in on sol 311 session in Figure 3b). The first arrivals have a dominant frequency of about 60 Hz (estimated from the dominant period of around 0.015 s; Figure 3b), which is lower than the dominant frequency of approximately 100 Hz observed during analog experiments on Earth (Kedar et al., 2016) likely due to the different environments. The signal‐to‐noise ratio measured as the ratio of the total energy within 50‐ms time windows before and after the first‐arrival onset time shows only minor variations over all sessions (±4 dB). At late recording times (*t* > 0.3 s), a strong, long‐lasting reverberation with a dominant frequency of around 25 Hz can be observed for most sessions (Figure 3a). It is suspected that this reverberation is a mechanical resonance but its origin has not yet been unambiguously identified (Hurst et al., 2021).

## 4 Seismic Data Analysis

### 4.1 P‐ and S‐Wave First‐Arrival Traveltime Picking

To characterize the first‐arriving energy, we performed a covariance‐based eigenanalysis of the three‐component particle motion within 4‐ms time windows around the first break (Greenhalgh et al., 2018) (see Appendix D for details on this polarization analysis). The eigenvector ${\hat{v}}_{1}$ associated with the largest eigenvalue reveals that the motion of the first‐arriving wave is oriented in the longitudinal (SEIS‐HP^3^ mole tip) direction at an azimuth of around 69° (Figure 2). The motion of the first‐arriving energy is thus consistent with the motion of a P‐wave traveling on the shortest path from the source to the receiver. Note that the observed motion within the P‐wave first‐arrival time window at the free surface is a combination of an incident P‐wave as well as a down‐going reflected P‐wave and a P‐S_*V*_‐converted wave, where S_*V*_ is the vertical transverse polarized S‐wave (see Appendix C). The direction of the apparent P‐wave particle motion is therefore not perfectly aligned with the actual propagation direction of the P‐wave.

**Figure 4:**
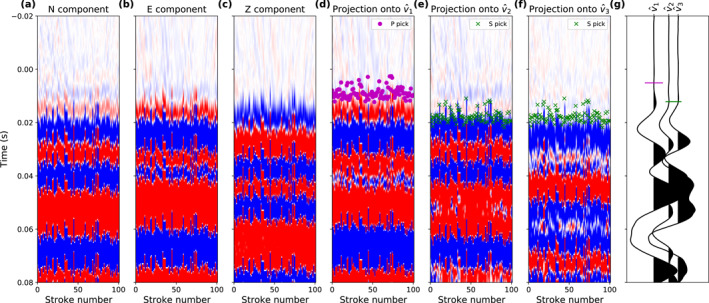
(a) North‐, (b) East‐, and (c) Vertical‐component seismic data recorded on sol 311. (d) Projection of panels (a–c) onto ${\hat{v}}_{1}$, which is assumed to be aligned with the first‐arrival P‐wave motion direction. The purple dots mark the manually picked P‐wave arrival times. (e and f) Projection of panels (a–c) onto ${\hat{v}}_{2}$ and ${\hat{v}}_{3}$, respectively, which are assumed to be free of P‐wave energy. The manually picked S‐wave arrival times are marked with green crosses. The same color scale as in Figure 3b is used for panels (a–f). (g) A single trace taken from panels (d, e, f) to better visualize the P‐ and S‐wave picks.

Rotating the East–North–Vertical recordings into a new coordinate frame with axes parallel to the eigenvectors ${\hat{v}}_{1}$–${\hat{v}}_{2}$–${\hat{v}}_{3}$ enhances particle motion interpreted as P‐wave energy in the ${\hat{v}}_{1}$ component. We then manually picked the P‐wave onset times for each hammer stroke on these rotated data. Figure 4 shows a data example of the component rotation and arrival time picking.

After the rotation that focuses all P‐wave energy in the ${\hat{v}}_{1}$ component, the ${\hat{v}}_{2}$ and ${\hat{v}}_{3}$ components contain the transverse polarized S_*V*_‐ and S_*H*_‐waves (Figures 4e and 4f). We then manually picked the onset times on the ${\hat{v}}_{2}$ and ${\hat{v}}_{3}$ components and interpret them as S‐wave first‐arrival times (green crosses in Figures 4e and 4f).

**Figure 5:**
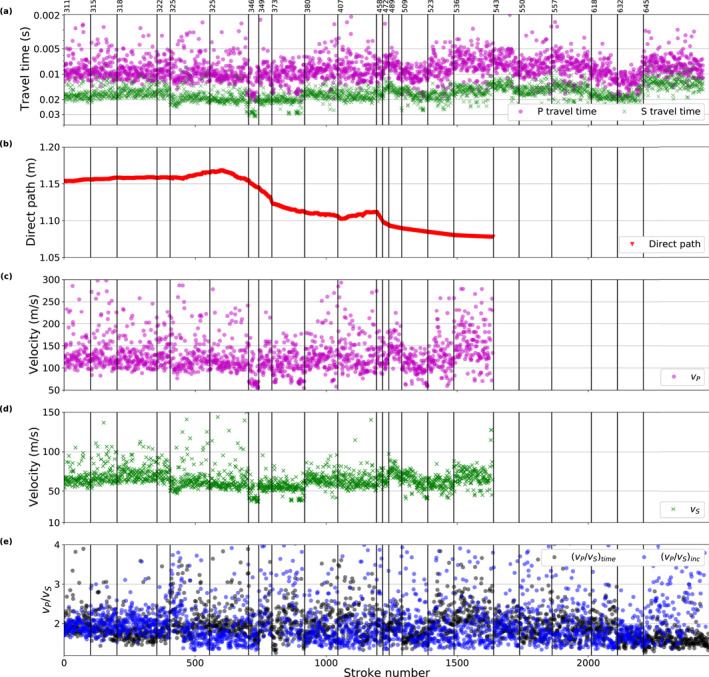
(a) First‐arrival P‐ (*t*_*P*_) and S‐wave (*t*_*S*_) traveltime picks for the hammer sessions conducted between sols 311 and 645. (b) Distance between the Heat flow and Physical Properties Package mole tip and Seismic Experiment for Interior Structure (*s*; see Equation 1 and Figure 1b). (c) Effective P‐ (*v*_*P*_) and (d) S‐wave velocity (*v*_*S*_) estimates based on the traveltimes and travelpath distances shown in panels (a and b), respectively. (e) ${\left(v_{P}/v_{S}\right)}_{\mathrm{time}}$ ratio estimates derived from *t*_*S*_/*t*_*P*_ using the traveltime data displayed in panels (a and b) plotted together with the incidence angle‐derived ${\left(v_{P}/v_{S}\right)}_{\mathrm{inc}}$.

**Figure 6:**
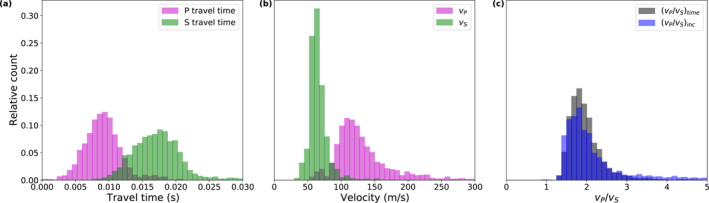
Histograms of (a) P‐ (*t*_*P*_) and S‐wave (*t*_*S*_) traveltime picks, (b) P‐ (*v*_*P*_) and S‐wave velocity (*v*_*S*_) estimates, and (c) ${\left(v_{P}/v_{S}\right)}_{\mathrm{time}}$ and ${\left(v_{P}/v_{S}\right)}_{\mathrm{inc}}$ ratios for hammer sessions conducted on sols 311–645. The *y*‐axis ticks plotted in panel (a) apply also to panels (b and c).

From a total of 2,461 recordings, we picked 2,438 P‐wave arrival times (*t*_*P*_) from which we selected those data that lie between the 2.5% and 97.5% quantile to exclude outliers (Figure 5a). The selected *t*_*P*_ picks range from 4.0 to 16.5 ms, with 50% of the data being within 7.3 and 10.6 ms (Figure 6a). A total of 2,438 S‐wave arrival times (*t*_*S*_) could be picked from the same recordings, ranging from 10.8 to 25.9 ms in the 2.5%–97.5% quantile range, with 50% of the data being within 15.6 and 19.7 ms (Figures 5a and 6a).

**Figure 7:**
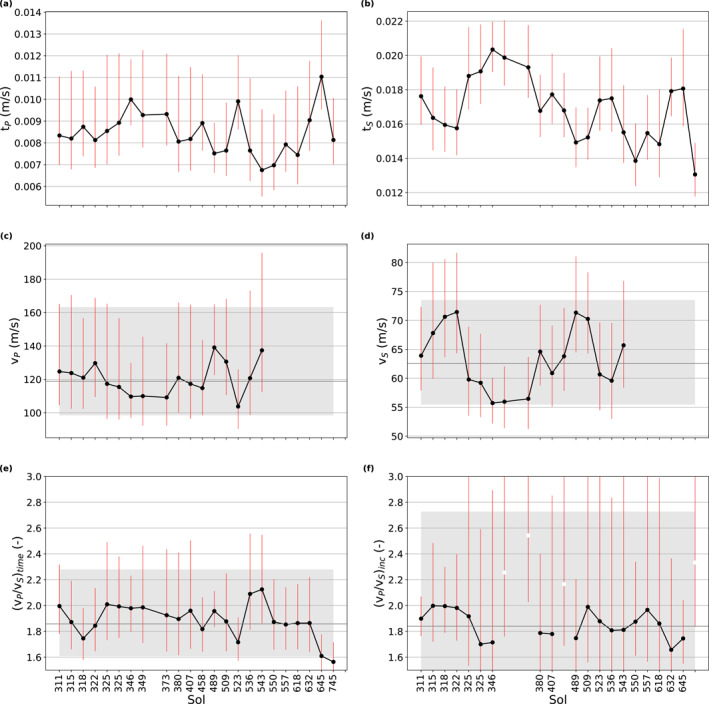
For each analyzed session, the mode (black dots) and 68.3% confidence intervals (red lines) of the log‐normal distributed data sets are shown (data within 2.5% and 97.5% quantiles). (a) *t*_*P*_, (b) *t*_*S*_, (c) *v*_*P*_, (d) *v*_*S*_, (e) ${\left(v_{P}/v_{S}\right)}_{\mathrm{time}}$, and (f) ${\left(v_{P}/v_{S}\right)}_{\mathrm{inc}}$. The white dots in panel (f) mark sessions that were excluded from the further analysis due to their large scatter. In panels (c–f), the horizontal dark gray lines and the light gray bar show the mode and 68.3% confidence interval of the entire data set, respectively (see Table 3 for values).

Both the P‐ and S‐wave traveltimes show a significant scatter within and in between sessions as visible in the histograms of the entire data set shown in Figure 6a and session‐wise plots of the traveltime variations (Figures 7a and 7b). The traveltimes show no significant correlation with distance, depth, or time/session. While the scatter within the sessions is similar for all sessions and for both P‐ and S‐traveltimes (i.e., 68.3% of the data are within −1.3 to 2.7 ms around the mode of the session; red bars in Figures 7a and 7b), the session's modes differ by up to 11 and 21 ms for P‐ and S‐traveltimes, respectively (black dots in Figures 7a and 7b). Variations of the modes between sessions are to some part due to changes in the length of the travelpath between the moving mole and SEIS. The traveltime variations within sessions are relatively similar for *t*_*P*_ and *t*_*S*_ pointing to a common source of the scatter for both *t*_*P*_ and *t*_*S*_. One source of error could come from the manual phase picking. We investigated the picking uncertainty by letting multiple people independently pick the same event and found a P‐wave traveltime variability of 0.031 ms. This picking uncertainty is small compared to, for example, the observed traveltime scatter within the sessions.

### 4.2 Velocity and *v*_*P*_/*v*_*S*_ Ratio Estimation From the Traveltime Data

To compute effective P‐ (*v*_*P*_) and S‐wave (*v*_*S*_) velocities, we assumed that the tip of the mole acted as the seismic source and divided *s* (Equation 1) by *t*_*P*_ and *t*_*S*_ (Figures 5a and 5b). Because no depth measurements were available for the last six sessions (sols 543–645), no *v*_*P*_ and *v*_*S*_ values could be computed for these sessions. Velocity estimates and corresponding histograms are shown in Figures 5c and 6b, respectively.

A total of 1,518 effective P‐ and S‐wave velocity estimates lie within the 2.5%–97.5% quantile and follow a log‐normal distribution (e.g., Limpert et al., 2001) with a mode and 68.3% confidence interval of $119_{-21}^{+45}$ m/s and $63_{-7}^{+11}$ m/s for P‐ and S‐waves, respectively. Because the traveltime scatter is similar in magnitude for *t*_*P*_ and *t*_*S*_ (see Figures 7a and 7b), the *v*_*P*_ estimates have a larger relative error compared to the *v*_*S*_ estimates (i.e.,%−21+45and%−7+11 for *v*_*P*_ and *v*_*S*_, respectively).

Assuming that both P‐ and S‐waves traveled along the same path, we computed the *v*_*P*_/*v*_*S*_ ratio for all 2,271 traveltime pairs from *t*_*S*_/*t*_*P*_. The resultant ${\left(v_{P}/v_{S}\right)}_{\mathrm{time}}$ ratio estimates have a mode and 68.3% confidence interval of $1.96_{-0.25}^{+0.42}$ (Figures 5d and 6c; Table 3).

**Table 3:** Velocity and *v*_*P*_/*v*_*S*_ Ratio Estimates Derived From the Traveltime $\left({\left(v_{P}/v_{S}\right)}_{\mathrm{time}}\right)$ and Amplitude $\left({\left(v_{P}/v_{S}\right)}_{\mathrm{inc}}\right)$ Data

Parameter	Mode and uncertainty bounds	Number of data points
*v*_*P*_ (m/s)	$119_{-21}^{+45}$	1,518
*v*_*S*_ (m/s)	$63_{-7}^{+11}$	1,518
${\left(v_{P}/v_{S}\right)}_{\mathrm{time}}$ (−)	$1.86_{-0.25}^{+0.42}$	2,271
${\left(v_{P}/v_{S}\right)}_{\mathrm{inc}}$ (−)	$1.84_{-0.35}^{+0.89}$	1,912

*Note.* Values correspond to the mode and 68.3% confidence interval of the log‐normal distributions (e.g., Limpert et al., 2001) estimated after exclusion of values outside the 2.5%–97.5% quantile range.

### 4.3 P‐Wave Incidence Angle‐Based *v*_*P*_/*v*_*S*_ Ratio Estimation

The incidence angle of a P‐wave observed at the free surface depends on the local elastic parameters below the receiver location (see also Appendix C). The apparent P‐wave incidence angle, therefore, offers an alternative observation independent of traveltime that provides constraints on the near‐receiver *v*_*P*_/*v*_*S*_ ratio. Svenningsen and Jacobsen (2007) and Edme and Kragh (2009) proposed techniques to exploit the fact that an incoming P‐wave interferes with the down‐going reflection and conversion at the solid‐air interface resulting in an observed apparent P‐wave incidence angle *θ*_*app*_ that is related to the true incidence angle *θ*_*P*_ as follows:

(2) $${\left(\frac{v_{P}}{v_{S}}\right)}_{\mathrm{inc}}=\frac{\sin\left(\theta_{P}\right)}{\sin\left(\frac{1}{2}\theta_{\mathrm{app}}\right)}.$$

Using an eigendecomposition of the three‐component waveform covariance matrix computed for a 7‐ms time window around the picked P‐wave traveltime, we estimated *θ*_*app*_ from the P‐wave first‐arrival polarization. Assuming that *θ*_*P*_ = 73° (average incidence angle from the HP3‐mole—SEIS geometry; see Figure 1b), a total of 2,461 incidence angle‐derived ${\left(v_{P}/v_{S}\right)}_{\mathrm{inc}}$ ratio estimates were calculated (Figures 5d and 6c).

The values from sessions of sols 349, 373, 458, and 645 show a large spread in arrival time (see Figure 7f) likely due to significant mole motion and/or significant mole dip that resulted in malfunctioning of the HP^3^ trigger. Excluding sessions 349, 373, 458, and 645 and using values with the 2.5% and 97.5% quantiles we find a ${\left(v_{P}/v_{S}\right)}_{\mathrm{inc}}$ ratio estimate of $1.84_{-0.35}^{+0.89}$ (Table 3), which is in reasonable agreement with the ${\left(v_{P}/v_{S}\right)}_{\mathrm{time}}$ ratio estimate of $1.86_{-0.25}^{+0.42}$.

We interpret the fact that ${\left(v_{P}/v_{S}\right)}_{\mathrm{inc}}$, which was derived independently of any clock‐time processing and traveltime picking, is close to ${\left(v_{P}/v_{S}\right)}_{\mathrm{time}}$ as an indication that the traveltimes are not contaminated by a significant time bias. A detailed analysis of a potential time bias impact on *v*_*P*_/*v*_*S*_ due to a systematic error in either both or only one of *t*_*P*_ and *t*_*S*_ revealed that such a time bias is maximum 0.9 ms and hence insignificant considering all other uncertainties (see Appendix E for an in‐depth discussion of a time‐bias impact).

## 5 Discussion

### 5.1 Validation of the Wavefield Separation for Recordings in the Near‐Field With Numerical Experiments

Given a P‐wave velocity of around 119 m/s and a dominant frequency of 60–120 Hz, the ratio of the travelpath to the dominant wavelength ranges from 0.6 to 1.2 m, which means that SEIS was located in the near‐field region of the seismic source. In the near‐field, the observed particle motion represents the combination of the P‐ and S‐wave far‐field components and a near‐field component, where the P‐wave and near‐field components arrive together first (Aki & Richards, 2009). In terms of polarization, the near‐field is composed of longitudinal and transverse motions. Representing the mole by a single force source and following Lokmer and Bean (2010), the near‐field term decays with distance as *r* ^−2^ for distances greater than half the dominant P‐wave wavelength.

Our traveltime interpretation after the polarization‐based wavefield separation is based on the assumption that the P‐ and S‐wave particle motions can be fully separated by three‐component rotation and the P‐ and S‐wave first arrivals are the first motions observed on the corresponding components (see Appendix D). While the traveltime of the near‐field first arrival corresponds to *t*_*P*_ (Aki & Richards, 2009), our *t*_*S*_ pick could be affected by near‐field components arriving before the true S‐wave first arrival.

With the motivation to assess the quality of our wavefield separation applied to near‐field data, we performed a 2D full‐wavefield simulation using a spectral element solver (Salvus; Afanasiev et al., 2019). We computed the seismic wavefield recorded at the free surface on the top of a homogeneous half‐space (*v*_*P*_, *v*_*S*_, and density values of 120 m/s, 60 m/s, and 1,300 kg/m^3^, respectively). We simulated seismic data generated by a 20°‐tilted force source at a depth of 0.32 m, resembling the mole at one of the early sessions. The source time function used was a Ricker wavelet with a dominant frequency of 60 Hz.

**Figure 8:**
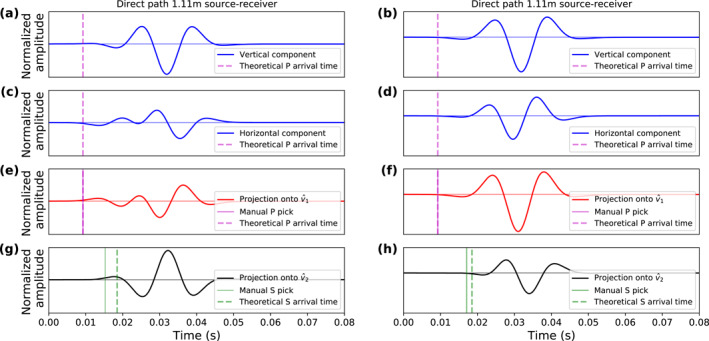
Synthetic data example computed for an 20°‐inclined force source in a 2D homogeneous half‐space and recorded at the free surface at 1.1 m distance to the left and right of the source (left panel: source points toward receiver). (a and b) Simulated vertical‐component recording. (c and d) Simulated horizontal‐component recording. (e and g) Projection of panels (a and c) onto the ${\hat{v}}_{1}$ and ${\hat{v}}_{2}$ components. (f and h) Projection of panels (b and d) onto the ${\hat{v}}_{1}$ and ${\hat{v}}_{2}$ components. The pink and green vertical lines show the true (dashed line) and manual (solid line) *t*_*P*_ and *t*_*S*_ picks.

We analyzed the simulated wavefield recordings for two different source‐receiver orientations to study the impact of the radiation patterns. Figures 8a and 8c show the vertical and horizontal wavefield components recorded with the inclined force source pointing toward the receiver at a source‐receiver distance of 1.1 m, while the vertical and horizontal wavefield components recorded at the same distance but on the opposite side of the source are displayed in Figures 8b and 8d. Following the polarization‐based wavefield separation outlined above, we rotated the data into a P‐ and S‐wavefield (i.e., ${\hat{v}}_{1}$ and ${\hat{v}}_{2}$ components) and picked the arrival times. The P‐wave first arrivals are clearly visible in ${\hat{v}}_{1}$ components and can accurately be picked at the correct times (Figures 8e and 8f).

The wavefield after projection onto the ${\hat{v}}_{2}$ components, however, shows near‐field term energy before the S‐wave arrival marked by the dashed lines in Figures 8g and 8h that can be misinterpreted as S‐wave arrival. The near‐field term is more pronounced in Figure 8g, which illustrates that the amplitude of near‐field term depends on the radiation pattern. The near‐field term leads to a tendency to picking *t*_*S*_ too early and, hence, to overestimate *v*_*S*_ and underestimate *v*_*P*_/*v*_*S*_. Because the two independently derived *v*_*P*_/*v*_*S*_ ratios from traveltimes ${\left(v_{P}/v_{S}\right)}_{\mathrm{time}}$ and apparent incidence angles ${\left(v_{P}/v_{S}\right)}_{\mathrm{inc}}$ are very close for the HP^3^ hammering seismic data measured on Mars, we assume that a potential time bias contaminating *t*_*S*_ must be small (see Appendix E for a discussion of the time bias).

### 5.2 Regolith Elastic Moduli

Assuming a density of 1,200 kg/m^3^ (Spohn et al., 2021), the *v*_*P*_ and *v*_*S*_ values with calculated uncertainties of $119_{-21}^{+45}$ m/s and $63_{-7}^{+11}$ m/s translate into a bulk, shear, and Young's modulus and a Poisson's ratio of $7.79_{-1.55}^{+1.60}$ MPa, $4.47_{-0.83}^{+2.00}$ MPa, $11.48_{-2.23}^{+5.91}$ MPa, and $0.28_{-0.051}^{+0.12}$, respectively (Table 4). When interpreting these values, one should keep in mind that they reflect values for a homogeneous volume and were derived from elastic waves with a dominant frequency of around 60 Hz. Consequently, the values from our study may be very different from static measurements to characterize the regolith material in terms of elastic moduli.

**Table 4:** Elastic Moduli Derived From the Seismic Velocity Estimates and Assuming a Density of 1,200 kg/m^3^ (Spohn et al., 2021)

Elastic moduli	Value
Bulk modulus (MPa)	$7.79_{-1.55}^{+1.60}$
Shear modulus (MPa)	$4.47_{-0.83}^{+2.00}$
Young's modulus (MPa)	$11.48_{-2.23}^{+5.91}$
Poisson's ratio (−)	$0.28_{-0.051}^{+0.12}$

Nevertheless, the elastic moduli derived from the traveltimes are in good agreement with estimates obtained in other studies. Spohn et al. (2021) derived shear strength estimates from the mole penetration resistance that correspond to a shear modulus range of 2–12 MPa, which agrees well with our estimate of $4.47_{-0.83}^{+2.00}$ MPa. Young's modulus estimates derived by Lognonné et al. (2020) from the resonance of the SEIS leveling system at around 51 Hz provide a value of about 46.8 MPa at the pressure of the SEIS mass of 8 kg, which corresponds to around 78 cm depth following the pressure dependence proposed by Morgan et al. (2018) and assuming a regolith density of 1,200 kg/m^3^. Converting this value to the mean depth between SEIS at the surface and the mole tip at depth of 16 cm results in a Young's modulus of around 18 MPa. Stott et al. (2021) derived an estimate of Young's modulus from the forcing of the lander in the frequency range of 0.1–0.9 Hz (assuming a density of 1,300 kg/m^3^ and a Poisson's ratio of 0.25). Converting the values from lander‐overload to surface‐pressure conditions provides a Young's modulus range of 30–40 MPa. The larger moduli found by Stott et al. (2021) may be due to the assumption of different density and Poisson's ratio values but could also be an effect of the lower frequency contents of the analyzed seismic data in the leveling system and lander resonance studies and, hence, the larger volume related to the effective moduli observations. However, all estimates come with a significant uncertainty and any differences should be discussed with care.

### 5.3 Geological Interpretation

The traveltime‐derived seismic velocities likely represent effective velocities averaged over a volume between the HP^3^ mole and SEIS with a suspected dimension on the order of several 10's of cm to 1 m cubed. The low velocities of $119_{-21}^{+45}$ and $63_{-7}^{+11}$ for *v*_*P*_ and *v*_*S*_, respectively, are compatible with a regolith layer dominated by mostly unconsolidated sand‐sized particles with a low density as observed from thermal inertia, thermal conductivity, and visual analysis of the soils around the lander (e.g., Golombek, Kass, et al., 2020; Grott et al., 2021).

A model of around 30 cm of the topmost regolith based on all observations from HP^3^‐mole and robotic arm operations as well as other geophysical and geological data consists of an approximately 1 cm thick dust layer at the surface, followed by duricrust about 20 cm thick above a 10 cm sand layer that transitions at around 30 cm depth into a sand‐gravel mixture (Spohn et al., 2021). This layering is too fine to be resolved with the recorded seismic traveltimes and the final velocity estimates found in this study likely represent an effective velocity for the entire stack of sand layers.

Thickness estimates of the mostly sandy regolith have been deduced from fresh 30–60 m diameter craters with nonrocky ejecta found in the vicinity of the InSight landing site suggesting a regolith layer about 3 m thick at the landing site (Golombek et al., 2017). The topmost meter of the regolith layer, for which our velocity estimates are representative, is most likely finer‐grained than at deeper levels as small impacts rather break up shallow material while only less frequent large impacts can penetrate to larger depths (Golombek, Warner, et al., 2020). The seismic velocities likely increase with depth, primarily governed by pressure within the topmost fine‐grained layer (Morgan et al., 2018).

Comparisons of the prelanding predicted low seismic regolith velocities on Mars with terrestrial soil and planetary regolith studies have extensively been discussed in Morgan et al. (2018). Similar low P‐wave velocities of 100–120 m/s have been observed during laboratory tests with different Martian regolith simulants and low overburden pressure (Delage et al., 2017). For the Moon, active source (e.g., Cooper et al., 1974) and passive (e.g., Sens‐Schönfelder & Larose, 2010) seismic experiments from Apollo 14, 16, and 17 as well as laboratory studies on lunar regolith samples (Johnson et al., 1982) found P‐wave velocities in the range of around 100–125 m/s at, or close to, the surface. Published lunar S‐wave velocities at the surface range between around 30 and 60 m/s (e.g., Dal Moro, 2015; Larose et al., 2005; Tanimoto et al., 2008), and reported Poisson's ratios range between 0.23 and 0.43 (e.g., Larose et al., 2005). Interestingly, these Poisson's ratios are generally higher than the predicted value for the InSight landing site that was estimated prelanding (i.e., 0.22 by Morgan et al., 2018) but agree reasonably well with the Poisson's ratio of 0.31 found in this study.

## 6 Conclusions

The recording of HP^3^ hammering signals using InSight's seismometer in order to constrain the regolith seismic velocities marks an opportunistic experiment. InSight's instrument suite was primarily designed for different purposes (i.e., thermal measurements at depth and the recording of marsquakes) and key changes that needed to be implemented to prepare the InSight hardware for a high‐resolution near‐surface seismic experiment were as follows: (a) The determination of sufficiently accurate source times, (b) the high‐resolution reconstruction of the broadband seismic hammering signals beyond the nominal SEIS sampling frequency, and (c) the clock correlation at the highest possible accuracy. By implementing these changes, we were able to record high‐resolution seismic data during the hammering of the HP^3^ mole.

We found low seismic velocities of $v_{P}=119_{-21}^{+45}$ and $v_{S}=63_{-7}^{+11}$ m/s based on the analysis of P‐ and S‐traveltimes. A *v*_*P*_/*v*_*S*_ ratio that is consistent with these estimates was found by an independent analysis of the P‐wave incidence angle. The low velocity values are in good agreement with the observed low‐density regolith of unconsolidated fine sands at the InSight landing site.

The velocity values likely represent some average (or bulk) effective velocity of the volume around the mole tip at around 0.3 m depth and SEIS at the surface. The *v*_*P*_ and *v*_*S*_ values from our study can serve as constraints for the inversion of other seismic data to resolve the deep structure at the landing site (e.g., H/V, Rayleigh wave ellipticity, and compliance inversion). Furthermore, the near‐surface regolith velocities can help to study the coupling of SEIS and the InSight lander to the ground to assess the impact of the regolith on the seismic measurements.

## Figures and Tables

**Figure B1:**
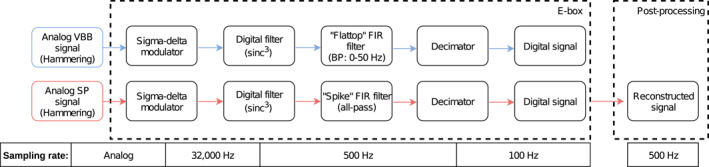
Seismic Experiment for Interior Structure down‐sampling flow of the very broad band (VBB) and short period (SP) data during HP^3^ hammering (Zweifel et al., 2021). *E‐box*: The analog signals recorded by the SP (red) and VBB (blue) are digitized by an analog‐to‐digital (ADC) converter at a sampling frequency of 32,000 Hz and represented by 24‐bit unsigned integers. Then, the ADC down‐samples the data to 500 Hz sampling frequency after the application of a third order sinc‐in‐time (sinc^3^) anti‐aliasing filter. Subsequently, the digitized data at 500 Hz are filtered with a Finite Impulse Response (FIR) filter and decimated to 100 Hz sampling frequency. *Postprocessing*: High‐frequency information of the SP data processed with an all‐pass FIR filter can be accurately recovered at an arbitrary sampling frequency (e.g., 500 Hz sampling frequency) by applying the reconstruction algorithm proposed by Sollberger et al. (2021).

**Figure C1:**
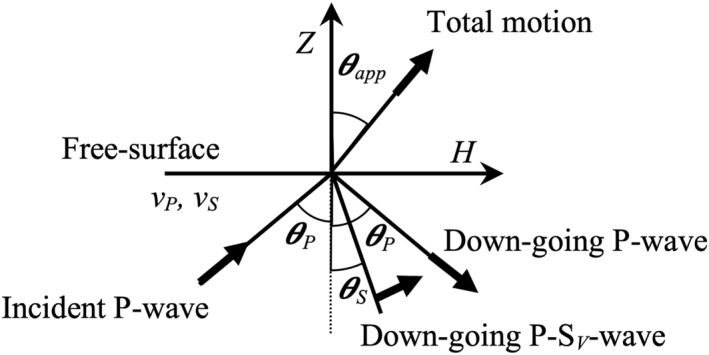
The total motion recorded by a receiver at the free surface of a solid elastic half‐space for an incident plane P‐wave is a combination of the incident P‐wave, reflected down‐going P‐wave and a down‐going P‐to‐S_*V*_ conversion. Arrows mark the direction of particle motion, where *Z* and *H* are vertical and horizontal components, respectively. The apparent angle *θ*_*app*_ is a function of the near‐surface velocities *v*_*P*_ and *v*_*S*_. The P‐ and S‐incident angles are also displayed with *θ*_*P*_ and *θ*_*S*_, respectively.

**Figure E1:**
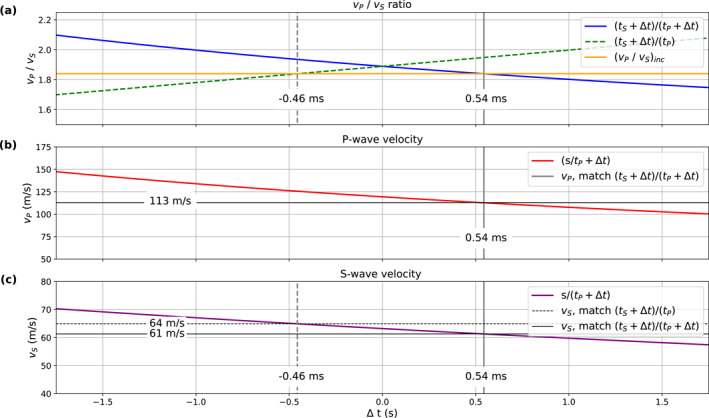
(a) The impact of time bias Δ*t* perturbing both the P‐ (*t*_*P*_) and S‐wave (*t*_*S*_) traveltime on *v*_*P*_/*v*_*S*_ = (*t*_*S*_ + Δ*t*)/(*t*_*p*_ + Δ*t*) ratio (blue curve) or only *t*_*S*_ and resulting in *v*_*P*_/*v*_*S*_ = (*t*_*S*_ + Δ*t*)/(*t*_*p*_) (green dashed line). Time shifts of either 0.54 ms or −0.46 ms are necessary to match ${\left(v_{P}/v_{S}\right)}_{\mathrm{inc}}=1.84$ (red line). (b) Dependence of *v*_*P*_ on Δ*t* added to *t*_*P*_. A Δ*t* of 0.91 ms to match ${\left(v_{P}/v_{S}\right)}_{\mathrm{inc}}=1.84$ results in *v*_*P*_ = 104 m/s. (c) Dependence of *v*_*S*_ on Δ*t* added to *t*_*S*_. A Δ*t* of 0.54 ms to match $\left(t_{S}+\Delta t\right)/\left(t_{p}+\Delta t\right)={\left(v_{P}/v_{S}\right)}_{\mathrm{inc}}=1.84$ results in *v*_*S*_ = 61 m/s, while a time bias affecting *t*_*S*_ only leads to *v*_*S*_ = 64 m/s. Calculations in panel (a–c) are based on an average travelpath of 1.1 m and unperturbed (i.e., Δ*t* = 0) velocities of 119 and 63 m/s for P‐ and S‐waves, respectively.

## Data Availability

Waveform data are available from the IPGP Datacenter and IRIS‐DMC (InSight Mars SEIS Data Service, 2019b). Seismic waveforms are also available from the NASA PDS (National Aeronautics and Space Administration Planetary Data System) (https://pds.nasa.gov/) (InSight Mars SEIS Data Service, 2019a). Visualizations were created with Matplotlib (Hunter, 2007) and data were processed with NumPy (Oliphant, 2007), SciPy (Virtanen et al., 2020), and ObsPy (Krischer et al., 2015). High‐rate seismic data from HP^3^ hammering obtained using the reconstruction algorithm designed by Sollberger et al. (2021) together with the trigger times in SEIS clock time are made available in a public repository at Sollberger et al. (2020). This is InSight Contribution Number 251.

## Appendix A HP^3^‐SEIS Clock Correlation

The individual electronic boards of Seismic Experiment for Interior Structure (SEIS) and Heat flow and Physical Properties Package (HP^3^) are not synchronized with one another and operate on different clocks. However, it is important to accurately link the two clocks to be able to connect HP^3^ trigger times with the seismic data recorded by SEIS. Since there is no direct link between the HP^3^ and SEIS clocks, the only way to convert the trigger times measured in HP^3^ clock time to SEIS clock time is via the spacecraft clock kernel (SCLK), which is part of the lander. Both clocks are occasionally correlated with the SCLK, which is therefore considered to be the reference clock.

The idea is to first convert the trigger times from HP^3^ clock time to SCLK and then convert the SCLK times to SEIS time. Once the trigger times are available in SEIS clock time, they are compatible with the time stamp of the seismic data. It is essential to convert the trigger times with high accuracy (e.g., tens of microseconds) as the traveltime of the seismic signals from source to receiver are extremely short. For example, a seismic wave traveling at 120 m/s (anticipated medium velocity obtained from Lognonné et al. (2020)) covering a distance of ∼1.1 m between source and receiver travels for ∼0.009 s. With the motivation to reduce the clock correlation errors to a negligible level, we target an accuracy of ∼1% of the traveltime, corresponding to ∼100 μs in our example.

The correlation between the HP^3^ and SEIS clocks and the SCLK is based on time correlation pairs. A time correlation pair is initiated by a pulse generated by the spacecraft at a known SCLK time and is recorded by the electronics of HP^3^ and SEIS both marking down the time stamp of the pulse arrival in their own clock time. Hence, a time correlation pair defines the relation between the SCLK and either the HP^3^ or SEIS clock. This relation is linear if both clocks do not suffer from a drift or if the drift is linear. Both the SEIS and HP^3^ clocks run slightly faster than the SCLK with a drift of around 1.5–4 and 1 ppm, respectively. Yet, only the HP^3^ clock drift is fairly linear in contrast to the SEIS clock drift, which is influenced by temperature changes in its electronic board. The drift causes an increase in time offset between the instrument clocks and the SCLK. The time offset between HP^3^ clock and SCLK is occasionally being reset following a science data request from the lander that directly equalizes the HP^3^ clock with the SCLK.

When the lander is on, the SCLK and the HP^3^ clock have a resolution of $\frac{1}{2^{16}}$ s and the SEIS clock counts at $\frac{1}{2^{10}}$ s. The SEIS clock and the SCLK exclusively write out a time correlation pair (hereinafter referred to as the SEIS‐SCLK pairs) every time the lander is turned on, whereas the sampling rate of time correlation pairs for the HP^3^ clock and the SCLK (hereinafter referred to as HP^3^‐SCLK pairs) is much higher. The HP^3^‐SCLK pairs are repeatedly sent when the lander is awake with samples every 14 s during hammering and samples every 120 s for hammer preheat and cool down phases. The time correlation pairs of both instrument clocks are available in the InSight housekeeping data.

To acquire the trigger times in SEIS clock time, we first convert the trigger times from HP^3^ clock time to SCLK. This is done by applying a linear interpolation as the internal drift of the HP^3^ clock is linear (i.e., constant increase in offset) and the HP^3^‐SCLK pairs are sampled densely. Then, we convert the SCLK times to SEIS clock time also using a linear interpolation method. However, the error induced by applying a linear interpolation in the second step is significant due to (a) the nonlinear drift of the SEIS clock due to temperature fluctuations and (b) very large time intervals between the SEIS‐SCLK pairs (e.g., up to 8 hr).

Figure A1a shows the two mentioned complications for the first hammer session conducted on sol 92. We observe that the two closest SEIS‐SCLK pairs are separated by 8 hours. This extensive interval period in combination with a nonlinear clock drift of SEIS effects the accuracy of the converted trigger times obtained from linear interpolation. The response of the clock drift on temperature changes influences the outcome of the trigger time converted to SEIS clock time. As the response relation of the clock drift of SEIS is unknown, we cannot accurately convert the trigger times from SCLK to SEIS time. As an example, in Figure A1a we show the trigger times converted from SCLK to SEIS time for a linear and a quadratic response, showing significant differences in their estimated trigger time.

We opt to reduce the error obtained by converting the trigger times from SCLK to SEIS clock down to a hundred microseconds in order to gain high precision information on the trigger time. The error induced by the linear interpolation between the SCLK and SEIS clock time is predominantly caused by the large interval length between the SEIS‐SCLK pairs (as shown in Figure A1a). To quantify the error obtained from the nonlinear drift of the SEIS clock, we assume that the SEIS temperature remains in the observed range from 0°C till 25°C (Zweifel et al., 2021). Then, the SEIS clock drift as a function of temperature changes at a maximum rate of 1 ppm/5°C. The highest gradient of the crystal temperature that has been observed is 2.5°C/3.5e3 s. Therefore, the maximum change in drift speed between the SEIS clock and the SCLK that could occur is 1 ppm/7e3 s and assuming that there is no offset at the start, we can define the drift (*d*(*t*)) as follows:

(A1)
d(t)=t⋅α,

where *t* is the time and *α* = 1 ppm/7e3 s. Then, the maximum difference between SEIS clock and SCLK (Δ*t*_max_) is as follows:

(A2)
$$\Delta t_{\max}\left(t\right)=\int d\left(t\right)\cdot dt$$

(A3)
$$=\frac{1}{2}\cdot t^{2}\cdot \alpha ,$$

To linearly estimate the time difference, we use the following equation:

(A4)
$$\Delta t_{\mathrm{lin}}\left(t\right)=\frac{1}{2}\cdot t\cdot \Delta t_{\mathrm{int}}\cdot \alpha ,$$

where Δ*t*_int_ is the time interval between the time correlation pairs. The largest time interval recorded between the SEIS‐SCLK intervals during hammering was up to 29,797 s on sol 94 (see Table A1). The error (*ϵ* = Δ*t*_*lin*_ − Δ*t*_max_) from linearly interpolating the trigger times in such a large time interval reaches a maximum of 0.0159 s at the middle of the interval (i.e., at *t* = 14,898.5 s). This error is beyond the resolution of the SEIS clock (∼1 ms) but reduces rapidly when the interval between the time correlation pairs decreases. Consequently, it also grows rapidly when the interval length increases as we observe in Figure A1a.

**Table A1:** The Maximum Time Interval Between the Seismic Experiment for Interior Structure (SEIS)‐Spacecraft Clock Kernel (SCLK) Correlation Pairs Measured During the Hammer Sessions

Hammer session	Maximum SEIS‐SCLK pair interval (hh:mm:ss.ms)	Drift error: *ϵ* (s)
sol 92	7:03:39.749	1.15e−2
sol 94	8:16:37.995	1.59e−2
sol 118	3:18:40.179	2.54e−3
sol 158–sol 632	0:00:50.000	4.64e−8

*Note.* The drift error defines the maximum error obtained from applying a linear interpolation between the SEIS‐SCLK correlation pairs to convert the trigger times from SCLK to SEIS clock time.

Reducing the interval length between SEIS‐SCLK pairs (Δ*t*) below 7,500 s is sufficient to obtain an error below the resolution of the SEIS clock. However, once the error is reduced below the resolution of the SEIS clock, the resolution itself is the principle component of the error. Then, the error is mostly dictated by the drift of the SEIS clock, which has a maximum of 4 ppm. As we aim to reduce the error down to a hundred microseconds, the time interval between the SCLK‐SEIS pairs is required to be further reduced to 50 s: $\frac{100\mu s}{0.5\cdot 4ppm}=50$ s.

For the first three hammer sessions (on sol 92, 94, and 118) the time interval between the time correlation pairs was very large due to unawareness of trigger time inaccuracies caused by interpolation. After realizing this, during all hammer sessions that followed, an additional command was sent to the spacecraft prior to hammering to set a fixed time interval of 50 s between SEIS‐SCLK synchronization pairs. Figure A1b shows the result of the linearly interpolated trigger times of the hammer session on sol 311, where the SEIS‐SCLK pairs are sampled every 50 s. In Table A1 we provide an overview of the maximum error caused by the SEIS‐SCLK drift together with the time interval between the correlation pairs for the conducted hammer sessions. For all hammer sessions later than session 118 the clocks can be synchronized with a resolution below 4.65e−8 s (Table A1), which meets our target accuracy of ∼1% of the expected traveltime. For comparison, independent and uniformly distributed HP^3^ and SEIS clock time quantization errors of 1/600 and 1/1,024 s sum to a trapezoid distribution with a standard deviation of around 0.6 ms.

## Appendix B Increasing Temporal Resolution

On Mars, the recorded analog signal is digitized and down‐sampled to a maximum rate of 100 sps in order to ensure preservation of all data in the limited onboard memory between downlinks to Earth (Lognonné et al., 2019). In the nominal setting of the very broad band (VBB) and short period (SP), the digitized data are passed through a finite impulse response (FIR) filter with a cutoff frequency at 50 Hz to avoid aliasing in the 100 sps data product.

Prior to hammering, a command is sent to the lander that loads different FIR filters for both the VBB and SP acquisition flow (Figure B1). The so called “flattop” FIR filter used for the VBB sensor during hammering has a different slope above the cutoff filter to avoid clipping of the high‐amplitude hammer signal. The SP recorded data are passed through an all‐pass FIR filter, the so‐called “spike” filter, during hammering and the reconstruction algorithm of Sollberger et al. (2021) is applied after the data is downlinked to Earth (Figure B1). Sollberger et al. (2021) extensively validated the reconstruction algorithm with synthetic data (see Section 4; Figure 6 in Sollberger et al. (2021)).

The SEIS acquisition control that includes digitizing, FIR filtering, and decimating introduces a certain delay in the seismic signals that needs to be accounted for when analyzing the data. Table B1 shows the delay introduced by each of the discussed FIR filters for data down‐sampled to 100 sps.

**Table B1:** Band‐Pass Coverage and Filter Delays for the Various Finite Impulse Response Filters at 100 sps

Filter	BP (Hz)	Total delay (ms)
Nominal	0–50	233.6
Spike	All	237.6
Flattop	0–50	233.6

## Appendix C P‐Wave Recording at the Free Surface

The motion recorded by a receiver placed at the free surface is the composite motion of the incident as well as reflected and converted waves at the free surface (e.g., Aki & Richards, 2009). For an incoming P‐wave, the composite recorded motion is the combination of the incident P‐wave, a reflected P‐wave, and a P‐S_*V*_ wave (Figure C1). The angles of the incident and reflected P‐wave *θ*_*P*_ are equal as well as the horizontal slowness *p* is preserved for all arrivals:

(C1)
$$p=\frac{\sin\;\theta_{P}}{v_{P}}=\frac{\sin\;\theta_{S}}{v_{S}}.$$

The total (observed) motion horizontal and vertical component recordings are the sum of the corresponding components of the three waves. Expressing the apparent angle *θ*_*app*_ as the ratio of the total horizontal (*H*) to vertical (*Z*) motion ratio results in (e.g., Aki & Richards, 2009; Edme & Kragh, 2009; Greenhalgh et al., 1990) the following equation:

(C2)
$$\begin{array}{ccc} \tan\;\theta_{\mathrm{app}}= & \frac{H}{Z} & =\tan\left(2\theta_{S}\right)\\ \theta_{\mathrm{app}} & = & 2\theta_{S} \end{array}$$

Equation 2 is then readily found by rearranging Equation C1 and replacing *θ*_*S*_ by $\left(\frac{1}{2}\theta_{\mathrm{app}}\right)$ (Equation C2).

## Appendix D Polarization Analysis

The configuration of the three SP components *U*, *V*, and *W* is not fully orthogonal (Lognonné et al., 2019). We therefore project the data from the original *U*‐*V*‐*W* configuration onto the orthogonal North (*N*), East (*E*), and vertical (*Z*) system by solving the following linear equation system:

(D1)
$$\left(\begin{array}{c} U\\ V\\ W \end{array}\right)=\left(\begin{array}{ccc} -\sin\left(-89.9\right) & \cos\left(285.0\right)\cos\left(-89.9\right) & \sin\left(285.0\right)\cos\left(-89.9\right)\\ -\sin\left(0.0\right) & \cos\left(105.2\right)\cos\left(0.0\right) & \sin\left(105.2\right)\cos\left(0.0\right)\\ -\sin\left(0.0\right) & \cos\left(345.3\right)\cos\left(0.0\right) & \sin\left(345.3\right)\cos\left(0.0\right) \end{array}\right)\cdot \left(\begin{array}{c} Z\\ N\\ E \end{array}\right),$$

where the elements of the rotation matrix are defined by the orientation of the *U*‐, *V*‐, and *W*‐axes.

We aim to separate the perpendicularly polarized P‐ and S‐ wavefields to confidently pick the P‐ and S‐wave first‐arrival times. To do so, we determine the polarization of the first‐arriving energy within a 4‐ms time window around the first‐arrival onset based on the assumption that the first‐arrival is a pure P‐wave arrival. We perform an eigendecomposition of the three‐component covariance matrix **C** computed for a time window of length *w* centered at *t*_*j*_:

(D2)
$$C\left(t_{j}\right)=\sum_{i=j-w/2}^{j+w/2}\left(\begin{array}{ccc} N\left(t_{i}\right)N^{T}\left(t_{i}\right) & N\left(t_{i}\right)E^{T}\left(t_{i}\right) & N\left(t_{i}\right)Z^{T}\left(t_{i}\right)\\ E\left(t_{i}\right)N^{T}\left(t_{i}\right) & E\left(t_{i}\right)E^{T}\left(t_{i}\right) & E\left(t_{i}\right)Z^{T}\left(t_{i}\right)\\ Z\left(t_{i}\right)N^{T}\left(t_{i}\right) & Z\left(t_{i}\right)E^{T}\left(t_{i}\right) & Z\left(t_{i}\right)Z^{T}\left(t_{i}\right) \end{array}\right)$$

In the case of a pure state, isolated arrival, the eigenvector $\left({\hat{v}}_{1}\right)$ associated with the largest eigenvalue of the covariance matrix **C** represents the main direction of polarization (Greenhalgh et al., 2018).

Subsequently, the azimuth (*ϕ*) and incidence angle (*θ*) of the dominant eigenvector ${\hat{v}}_{1}$ can be determined as follows:

(D3)
$$\tan\;\phi =\frac{{\hat{v}}_{1N}}{{\hat{v}}_{1E}}$$

(D4)
$$\tan\;\theta =\frac{\sqrt{{\hat{v}}_{1E}^{2}+{\hat{v}}_{1N}^{2}}}{\left|{\hat{v}}_{1Z}\right|}$$

The incidence angle *θ* can then be used to obtain the incidence angle‐based ${\left(v_{P}/v_{S}\right)}_{\mathrm{inc}}$ ratio.

Once the dominant polarization direction is determined, we can rotate the three component *N*–*E*–*Z* data into a new coordinate frame *V*_1_–*V*_2_–*V*_3_ with axes parallel to ${\hat{v}}_{1}$, ${\hat{v}}_{2}$, and ${\hat{v}}_{3}$, respectively using the rotation matrix *R*:

(D5)
$$\left(\begin{array}{c} V_{1}\\ V_{2}\\ V_{3} \end{array}\right)=R\left(\begin{array}{c} N\\ E\\ Z \end{array}\right),$$

where **R** is defined as follows:

(D6)
$$R\;=\left(\begin{array}{ccc} \cos\;\phi \cos\;\theta & -\cos\;\theta \sin\;\phi & \sin\;\theta \\ \sin\phi & \cos\;\phi & 0\\ -\cos\;\phi \sin\;\theta & \sin\;\theta \sin\;\phi & \cos\;\theta \end{array}\right),$$

Assuming that the first arrival is a rectilinearly polarized P‐wave and ${\hat{v}}_{1}$ is aligned with the P‐wave motion, then the P‐wave energy is isolated in the *V*_1_ component, while the *V*_2_ and *V*_3_ components are P‐wave energy free and contain the transverse polarized S‐wave energy. Because the apparent P‐ and S‐wave polarization may not be exactly perpendicular (see Appendix C), some S‐wave energy may leak into the V_1_ component.

## Appendix E Impact of a Time Bias on Velocity Estimates

One potential issue of the HP^3^‐SEIS traveltime interpretation could be that a time bias Δ*t* contaminates one or both *t*_*P*_ and *t*_*S*_ (e.g., consistently early or late triggering due to an inaccurate trigger threshold; consistent bias in the traveltime picks). The incidence angle‐derived ${\left(v_{P}/v_{S}\right)}_{\mathrm{inc}}$ ratio estimates allow us to assess the reliability of the traveltime‐based velocity estimates because the ${\left(v_{P}/v_{S}\right)}_{\mathrm{inc}}$ ratio estimates were derived independent from the traveltimes based on the first‐arrival amplitudes.

A time bias applied to both *t*_*P*_ and *t*_*S*_ will affect *v*_*P*_/*v*_*S*_ = (*t*_*S*_ + Δ*t*)/(*t*_*P*_ + Δ*t*) such that the *v*_*P*_/*v*_*S*_ ratio will decrease for an increasing Δ*t* for a fixed travelpath (Figure E1a; assuming an average travelpath of 1.1 m, unperturbed traveltimes of 9.21 and 17.4 ms (Δ*t* = 0) and velocities of 119 and 63 m/s for P‐ and S‐waves, respectively). The Δ*t* needed to be applied to both *t*_*P*_ and *t*_*S*_ to match a given *a* = (*v*_*P*_/*v*_*S*_) is as follows:

(E1)
$$\Delta t_{1}=\frac{t_{S}-at_{P}}{\left(a-1\right)}.$$

For ${\left(v_{P}/v_{S}\right)}_{\mathrm{inc}}=$ 1.84, we find Δ*t*_1_ = 0.54 ms (Figure E1a), marking the time bias needed to make (*t*_*S*_ + Δ*t*_1_)/(*t*_*p*_ + Δ*t*_1_) match ${\left(v_{P}/v_{S}\right)}_{\mathrm{inc}}$. Because Δ*t*_1_ increases both traveltimes, both velocities decrease to *v*_*P*_ = 113 m/s and *v*_*S*_ = 61 m/s (Figure E1b and 1c). Δ*t*_1_ could be an error in the clock‐time processing affecting both *t*_*P*_ and *t*_*S*_ in the same way. The estimated drift error presented in Table A1 (for the hammer sessions on sol 158–sol 632) of Δ*t*_1_ = 4.64*e* ^−8^ ms results in velocity estimates of *v*_*P*_ = 119 m/s and *v*_*S*_ = 63 m/s, which shows that the uncertainty due to the clock‐time processing on the velocity estimates is negligible.

If only *t*_*S*_ is affected by a time bias, then the resultant *v*_*P*_/*v*_*S*_ = (*t*_*S*_ + Δ*t*)/*t*_*P*_ increases with Δ*t* (Figure E1a). The Δ*t*_2_ to match $a={\left(v_{P}/v_{S}\right)}_{\mathrm{inc}}=1.84$ is as follows:

(E2)
$$\Delta t_{2}=at_{P}-t_{S}=-0.46\;ms.$$

Consequently, *v*_*S*_ increases to *v*_*S*_ = 64 m/s (Figure E1c). One potential time bias effecting *t*_*S*_ only could be a consistent too early picking because of a contamination of the ${\hat{v}}_{2}$ and ${\hat{v}}_{2}$ components first arrivals by near‐field term energy (see Figure 8). However, if this was the case, then we would expect Δ*t*_2_ > 0. If only *t*_*P*_ was affected, we found Δ*t*_3_ = 0.25 ms and a resultant velocity of *v*_*P*_ = 116 m/s. Because all Δ*t* are small compared to other uncertainties (e.g., traveltime scatter within and between sessions, see Figure 7), we consider time biases as minor source of errors.
